# Generation of T follicular helper cells *in vitro*: requirement for B‐cell receptor cross‐linking and cognate B‐ and T‐cell interaction

**DOI:** 10.1111/imm.12834

**Published:** 2017-10-09

**Authors:** Anne Kolenbrander, Bastian Grewe, David Nemazee, Klaus Überla, Vladimir Temchura

**Affiliations:** ^1^ Department of Molecular and Medical Virology Ruhr‐University Bochum Germany; ^2^ Department of Immunology and Microbial Science The Scripps Research Institute La Jolla CA USA; ^3^ Institute of Clinical and Molecular Virology University Hospital Erlangen, Friedrich‐Alexander University Erlangen‐Nürnberg (FAU) Erlangen Germany

**Keywords:** cognate B cells, HIV, T follicular helper cells, virus‐like particles

## Abstract

The minimum requirements for *in vitro* modelling of natural CD4^+^ T‐cell differentiation into T follicular helper (Tfh) cells are still under investigation. We co‐cultured wild‐type and T‐cell receptor (TCR) transgenic CD4^+^ T cells from naive mice with dendritic cells and B‐cell receptor (BCR) transgenic B cells in the presence of HIV‐derived virus‐like particles containing matched B‐cell and T‐cell epitopes. This co‐culturing induced co‐expression of Tfh‐master regulator transcription factor BCL‐6 and CXCR5 in up to 10% of the wild‐type and up to 40% of the TCR‐transgenic CD4^+^ T cells. Phenotypic markers, production of interleukin‐21 and isotype switching of the B cells to IgG1 further indicated a helper function of the induced Tfh cells *in vitro*. Dendritic cells supported the generation of functional Tfh cells, but were unable to induce them without cognate B cells. Hence, our study presents a robust experimental system for efficient generation of functionally active Tfh cells *in vitro* and confirms the importance of cognate B‐ and T‐cell cross‐talk for the Tfh differentiation process.

**Abbreviations**
b12: b12HL mice
BCL‐6: B‐cell lymphoma 6 protein
BL6: C57BL/6J mice
CA: capsid protein p24 of HIV‐1
Env: envelope protein of HIV‐1
Exo: exosomes
Gag: group‐specific antigen of HIV‐1
HEL: hen egg lysozyme
HIV: human immunodeficiency virus
HKG: housekeeping gene
MA: matrix protein p17 of HIV‐1
OT2: H‐2b‐restricted MHC class II epitope of ovalbumin_323–339_
Pol: Pol polyprotein of HIV‐1
RLU/c: relative light units per second
Tfh: T follicular helper
VLPs: virus‐like particles
wt: wild‐type

## Introduction

T follicular helper (Tfh) cells are a separate lineage of T helper cells with distinct developmental and effector functions. Tfh cells provide cognate help for B cells and are important for the formation of germinal centres and subsequent long‐lived antibody responses. Despite remarkable heterogeneity of Tfh cells in function and phenotype, the expression of the master regulator transcription factor BCL‐6 is a common molecular hallmark of these cells.^1^, ^2^

To improve the efficacy of vaccines it is critical to understand the establishment of Tfh responses and the way to manage this establishment to reach an optimal humoral immunity. Generation of Tfh cells from naive (antigen unexperienced) CD4^+^ T cells *in vivo* is a complex process that requires contact with dendritic cells (DCs), B cells, signalling via T‐cell receptor (TCR), co‐stimulatory molecules and cytokines.^2^, ^3^ Current protocols for *in vitro* generation of Tfh‐like cells are restricted to TCR‐transgenic T cells that require sophisticated stimulation protocols.^4^, ^5^ However, a robust model for the generation of Tfh cells *in vitro* that avoids manipulation by exogenous cytokines and antibodies is still lacking.

By investigating the effect of virus‐like particles (VLPs) derived from human immunodeficiency virus (HIV) on the cross‐talk between B and T cells through intrastructural help, we observed accumulation of Tfh‐like cells *in vitro*. These cells were generated during the co‐culturing of B‐cell receptor (BCR) transgenic B cells together with wild‐type (wt) DCs and wt CD4^+^ T cells from antigen‐primed mice.^6^ In this co‐culture model, the efficient activation of BCR‐transgenic B cells specific for the hen egg lysozyme (HEL) was achieved by VLPs, containing an inner core of HIV‐Gag proteins surrounded by a lipid bi‐layer with repetitively anchored HEL at the outer surface of the particles (HEL‐VLPs).^7^ The CD4^+^ T cells were derived either from naive mice or from mice immunized against the HIV‐Gag core protein of the VLPs.^6^ Although, differentiation of T cells into Tfh‐like cells from Gag‐primed mice was highly efficient, reaching up to 18% of all CD4^+^ T cells in the co‐culture, approximately 10% of the T cells from non‐immunized naive mice had a Tfh‐like phenotype as well.^6^

Better understanding of the differentiation of non‐TCR transgenic T cells into Tfh cells *in vitro* can improve the development of novel vaccination strategies. Here, we explored the differentiation of primary wt CD4^+^ T cells into Tfh cells in the absence of any additional stimulation by exogenous cytokines in more detail. We also addressed the requirement for cognate epitope recognition by the B and T cells using BCR‐ and TCR‐transgenic lymphocytes. Both experimental settings revealed the requirement for cognate B‐ and T‐cell interactions, BCR cross‐linking and accessory role for DCs in the Tfh differentiation process *in vitro*.

## Materials and methods

### Mice

Mice were housed in singly ventilated cages at the animal facility of Medical Faculty, Ruhr‐University Bochum (Germany) in accordance with the national law. Mice were handled according to the instructions of the Federation of European Laboratory Animal Science Association. Six‐ to eight‐week‐old C57BL/6J (BL6) (Janvier, France), OT2 (in‐house breeding), SW‐HEL (in‐house breeding) and b12HL (b12) (in‐house breeding) mice were used.

### Production and characterization of VLPs and exosomes

To produce HEL‐VLPs, VLPs lacking HEL, and HEL‐containing exosomes HEK293T cells were transiently transfected with the HIV *gag/pol* expression plasmid Hgpsyn and/or an expression plasmid for membrane‐anchored HEL (pC‐HEL‐TM) using polyethylenimine as described in detail elsewhere.^7^ For Env‐VLP production the cells were transfected with the HIV *env* expression plasmid pConBgp140GCD and Hgpsyn.^6^ To produce VLPs with HIV‐Gag protein containing the H‐2b‐restricted MHC class II OVA_323–339_ peptide (OT2), the Hgpsyn‐OT2 plasmid was used instead of Hgpsyn during transfection. In the Hgpsyn‐OT2 plasmid the coding sequence for the OT‐2 epitope ISQAVHAAHAEINEAGR was inserted between the codons for the Gag amino acid DTGHSSQ and VSQNYPI at the C terminus of Gag's matrix domain encoded in the Hgpsyn by overlap‐extension PCR. The cleavage site of the viral protease between the matrix and capsid proteins was kept intact in the Hgpsyn‐OT2, leading to the processing of Gag comparable to that seen for Gag produced by the Hgpsyn (data not shown). VLPs and exosomes were concentrated and purified from the supernatant of transfected cells by ultracentrifugation through a 20% sucrose cushion 2 days after transfection.^6^, ^7^ Determination of HIV p24 Gag, HIV Env and HEL concentrations was performed with specific ELISAs as reported elsewhere.^6^, ^7^

### Cell isolation and purification

For the purification of DCs, single‐cell suspensions of BL6 mouse splenocytes were prepared as previously described.^8^ CD11c^+^ DCs were enriched by positive selection with anti‐CD11c magnetic beads (#130‐52‐001; Miltenyi Biotec, Bergisch Gladbach, Germany). Untouched CD4^+^ T cells were isolated from single‐cell suspensions of splenocytes and lymph node cells from naive (non‐immunized) BL6 and OT2 mice with the CD4^+^ T Cell Isolation Kit (#130‐104‐454; Miltenyi Biotec). Untouched B cells were isolated from single‐cell suspension of splenocytes and lymph node cells from naive BL6, SW‐HEL and b12 mice with the B‐Cell Isolation Kit (#130‐90‐862; Miltenyi Biotec). All isolations were performed according to the manufacturer's instructions. The resulting cells were routinely >98% pure.

### In vitro co‐culture experiments

Cells in R10 medium (RPMI‐1640 (Gibco, Gaithersburg, MD), supplemented with 10% fetal calf serum, 50 μm *β*‐mercaptoethanol, 10 mm HEPES buffer and penicillin‐streptomycin) were plated in U‐bottom 96‐well plates at a density of 2 × 10^5^ CD4^+^ T cells/well, the B cells were added in a ratio of 1 : 2 (B : T) and DCs in a ratio of 1 : 5 (DC : T) and incubated for 3 or 6 days in the presence of different stimuli. All VLPs were added at the following final concentrations: 50–100 ng/ml of HIV‐p24 Gag for all VLPs; 30–40 ng of HEL/ml for HEL‐VLPs and HEL‐Exo; 100 ng/ml of HIV‐gp120 for Env‐VLPs and monomeric gp120 protein; 100 ng/ml of soluble OT2 peptide. For the intracellular staining against interleukin‐21 (IL‐21), IL‐4 or interferon‐*γ* (IFN‐*γ*) 0·02 mm Monensin was added in some cultures on day 6 and the cells were incubated for another 6 hr before the analysis.

### FACS analyses

After co‐culture, the cells were collected and stained with anti‐B220, CD4, CXCR5, GL‐7, ICOS, PD‐1 and CD40L antibodies for surface expression and with anti‐BCL‐6, T‐bet, GATA3, IL‐21, IL‐4 and IFN‐*γ* antibodies for intracellular proteins. Antibodies were obtained from eBioscience (San Diego, CA) or BD Biosciences (San Jose, CA). Intracellular staining was performed by fixation with 2% paraformaldehyde and permeabilization with 0·5% saponin as described.^6^ The stained cells were analysed by flow cytometry using BD FACSCanto II. A dump channel was used to exclude auto‐fluorescent cells; doublets were discriminated using both FSC‐H versus FSC‐A and SSC‐H versus SSC‐W gating strategies (see Supplementary material, Figs S1 and S3).

### CFSE proliferation assay

The proliferation assay of OT2 CD4^+^ T cells in co‐culture with DCs was performed as described elsewere.^9^ Briefly, CD4^+^ T cells were labelled with 7·5 μm CFSE (Vybrant CFDA Cell Tracer kit; Invitrogen, Carlsbad, CA) and co‐cultivated with freshly isolated splenic DCs (from BL6 mice) for 64 hr at 37° in the presence of different VLP preparations in various concentrations, as described above. For the proliferation assay of co‐cultures with B cells, 2 × 10^5^ CFSE‐labelled CD4^+^ T cells from OT2 mice were co‐cultured with 1 × 10^5^ B cells from b12 or wt BL6 or SW‐HEL mice for 64 hr at 37° in the presence of different VLPs in the concentrations described above. CFSE dilution in CD4^+^ cells (see Supplementary material, Fig. S2) was analysed by flow cytometry.

### Reverse transcription quantitative PCR

For the quantification of IL‐21 mRNA in cell cultures, total RNA was extracted at days 4, 5 and 6 of culturing with the RNeasy Mini Kit (Qiagen, Hilden, Germany) according to the manufacturer's instructions. Relative quantities of mRNA for IL‐21 (sense 5′‐GCCAGATCGCCTCCTGATTA‐3′, antisense 5′‐CATGCTCACAGTGCCCCTTT‐3′) and housekeeping genes determined with Rotor‐Gene Q Platform (Qiagen) using SYBR Green PCR Master Mix. mRNA levels for each sample were normalized to housekeeping gene mRNA levels and presented as a relative increase (fold increase) compared with levels in controls. Each experiment was performed at least three times.

### Measurement of antibody levels in conditioned media

After 6 days, cell‐free supernatants of co‐cultures were analysed by ELISAs to detect immunoglobulin subclasses as described previously.^6^, ^7^

### Statistical analysis

Statistical analysis was performed with graph pad prism (Graph Pad Software, San Diego, CA).

## Results

### Minimal requirements for generation of wt BCL‐6^+^ CXCR5^+^ CD4^+^ T cells *in vitro*

To determine the minimal requirements for generation of Tfh cells *in vitro*, we co‐cultured untouched wt CD4^+^ T cells with (i) wt splenic DCs, or (ii) untouched BCR‐transgenic B cells from SW‐HEL mice, or (iii) wt DCs with SW‐HEL B cells all freshly isolated from naive animals. Antigenic stimulation was provided by HEL‐VLPs^7^ (see Discussion) (Table 1). As a control for the antigen‐specificity of the differentiation process we used VLPs that lack the B‐cell cognate antigen (VLPs in Table 1).

**Table 1:** Lentiviral virus‐like particle (VLP)/exosomal preparations used in the study

Abbreviations	Envelope proteins	Structure proteins
VLPs	No specific^a^	HIV‐Gag/Pol
HEL‐Exo	Membrane‐anchored HEL	No specific^a^
HEL‐VLPs	Membrane‐anchored HEL	HIV‐Gag/Pol
HEL‐OT2‐VLPs	Membrane‐anchored HEL	HIV‐Gag/Pol with OT2
Env‐VLPs	HIV‐Env	HIV‐Gag/Pol
Env‐OT2‐VLPs	HIV‐Env	HIV‐Gag/Pol with OT2

a Contain host proteins from the human HEK293T producer cell line.

**Figure 1:**
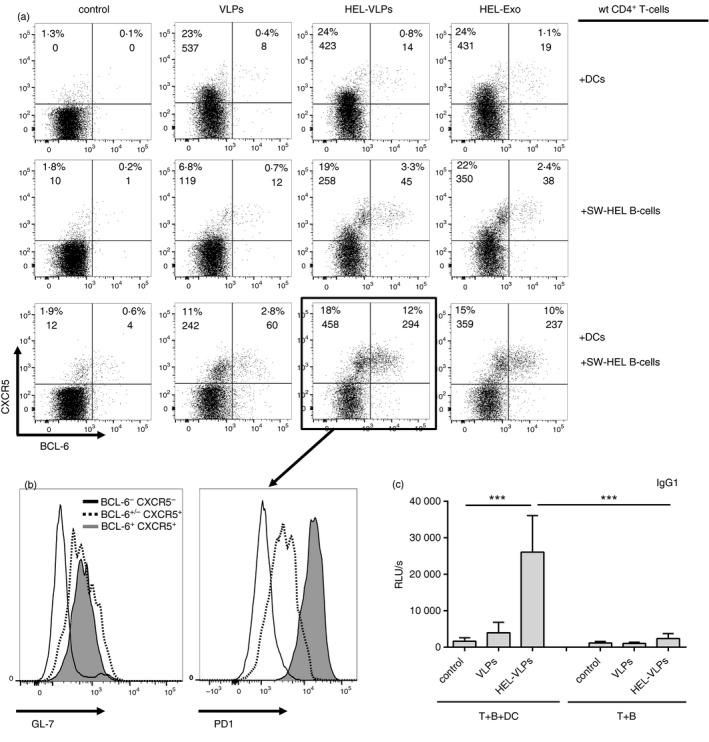
Generation of wild‐type (wt) follicular helper T (Tfh) ‐like cells with T‐helper function after 6‐day co‐culture experiments. CD4^+^ T cells from naive BL6 mice, B cells from naive SW‐HEL mice and/or splenic dendritic cells (DCs) from BL6 mice were co‐cultured in the presence of hen egg lysozyme–virus‐like particles (HEL‐VLPs), HEL‐Exo or control VLPs without surface HEL. After 6 days of incubation, cells were stained for surface CD4, CXCR5, GL‐7, PD1 and intracellular BCL‐6. The gating strategy is demonstrated in the Supplementary material (Fig. S1). Dot plots (a) and histograms (b) represent singlets of the non‐auto‐fluorescent CD4^+^ cells. (a) Numbers in the quadrants indicate percentages of the respective subpopulation among the CD4^+^ cells and cell counts per time package. (b) Histograms represent GL‐7 and PD1 surface expression on subpopulations of CD4^+^ cells from co‐culture with naive SW‐HEL B cells and DCs from BL6 mice in the presence of HEL‐VLPs. Each experiment was performed three times in duplicates. The data of one representative experiment are shown. (c) Supernatants from SW‐HEL B cells cultured over 6 days with CD4 T cells and +/− DCs (T+B+DC or T+B) in the presence of HEL‐VLPs or VLPs were analysed by ELISA for the presence of anti‐HEL IgG1. The results are presented as relative light units per second (RLU/s). The histograms represent the mean from three independent experiments (two samples each) ± standard deviation. ***p < 0·001; one‐way analysis of variance with Tukey's post test. The data from co‐cultures of CD4^+^ T cells, DCs, and SW‐HEL B cells (Fig. 1a, bottom row) have been reported previously.^6^

On day 3 of co‐culture, we could not detect induction of intracellular BCL‐6 expression in any experimental group (data not shown). Co‐culture of wt CD4^+^ T cells with DCs in the presence of both VLP variants up to day 6 induced expansion of CD4^+^ T cells expressing intermediate levels of CXCR5 and minimal induction of BCL‐6 expression (Fig. 1a, top row). Co‐culture of SW‐HEL B cells with wt CD4^+^ T cells and HEL‐VLPs led to further differentiation of CD4^+^ T cells mainly into cells with pre‐Tfh‐like phenotype (BCL‐6^+/−^ CXCR5^+^) and into few Tfh‐associated (BCL‐6^+^ CXCR5^+^) cells (Fig. 1a, middle row). We observed the strongest expansion of CD4^+^ T cells with Tfh‐associated phenotype (BCL‐6^+^ CXCR5^+^) when wt CD4^+^ T cells were co‐cultured together with both DCs and B cells in the presence of HEL‐VLPs (Fig. 1a, bottom row). More importantly, control VLPs without HEL only modestly stimulated the induction of Tfh‐like cells (up to 3%). To avoid an influence of HIV‐Gag or GagPol proteins present in the VLPs in the generation of the Tfh cells, we also stimulated the co‐cultures with exosomes containing HEL (HEL‐Exo), but lacking Gag and GagPol proteins (Table 1).^8^ This revealed a similar differentiation process of CD4^+^ T cells into Tfh cells by co‐culture with DCs and/or transgenic B cells as observed for HEL‐VLPs (Fig. 1a, right column HEL‐Exo). Our results suggest that a repetitive, membrane‐anchored antigen is sufficient for the activation of B cells through BCR, enabling these B cells to trigger Tfh differentiation.

### Phenotypic and functional characterization of *in vitro* generated wt BCL‐6^+^ CXCR5^+^ CD4^+^ T cells

PD1 serves as an important Tfh cell marker *in vivo* 10 and GL‐7 was identified as a specific surface marker of germinal centre Tfh cells.^11^ Almost all CD4^+^ BCL6^+^ CXCR5^+^ cells generated by co‐culture with DCs and SW‐HEL B cells in the presence of HEL‐VLPs were GL‐7 positive (Fig. 1b, left) and PD1^high^ (Fig. 1b, right), confirming their Tfh cell phenotype.

It is accepted that T‐dependent class‐switch recombination, even before germinal centre development, primarily mediated by Tfh cells.^2^ Previously, we have demonstrated that in the absence of T‐cell help, HEL‐VLPs can induce IgG3, but not IgG1 or IgG2c, isotype switching in cognate SW‐HEL B cells *in vitro*.^7^ Analysis of HEL‐specific immunoglobulin levels and isotypes in the cell‐culture medium at day 6 revealed isotype switching to IgG1 (Fig. 1c). Hence, the Tfh cells generated are able to trigger differentiation of naive B cells into IgG1 secreting cells and, therefore, can be considered to be functionally active Tfh cells.

### Processing and presentation of the OT2 epitope from the VLPs structure proteins to the CD4^+^ T cells is BCR‐dependent

**Figure 2:**
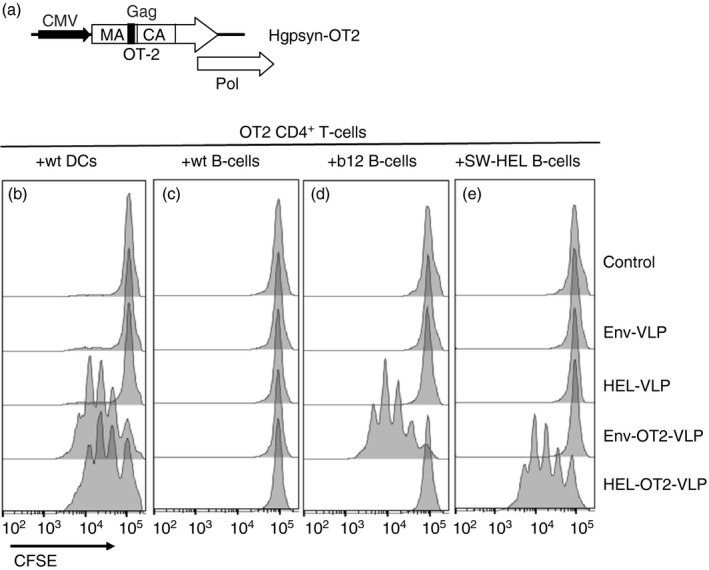
Design and characterization of HIV‐derived virus‐like particles (VLPs) containing the OT2 epitope. (a) Schematic representation of the Hgpsyn‐OT2 expression cassette. CMV, immediate early promoter of human cytomegalovirus; MA, matrix; CA, capsid; Pol, polymerase. (b–e) Untouched CD4^+^ T cells were isolated from naive OT2 mice, labelled with CFSE and co‐cultured with splenic (b) dendritic cells (DCs), or with B cells from (c) wild‐type (wt) BL6, (d) b12 and (e) SW‐HEL mice for 64 hr in the presence of the indicated VLPs (50 ng of p24 capsid/ml). CFSE fluorescence intensities of the singlet CD4^+^ cells for one of six representative experiments are shown. The gating strategy is presented in the Supplementary material (Fig. S2).

To explore whether recognition of the cognate epitope by the TCRs of the Tfh precursor cells is required for the generation of Tfh cells *in vitro*, we used CD4^+^ T cells from OT2 TCR‐transgenic mice and incorporated the OT2 epitope into the HIV‐Gag protein of the VLPs. We inserted the coding sequence for the OT2 epitope between the matrix and capsid domains of the codon‐optimized HIV‐gag/pol expression plasmid (Fig. 2a) as described previously.^9^ To further confirm our results obtained with the B cells from SW‐HEL BCR‐transgenic mice we used the B cells from b12 mice, another BCR‐transgenic mouse line expressing the broadly neutralizing antibody b12 that is specific to the HIV‐Env gp120 protein.^12^

First, we analysed the ability of DCs and B cells to present the OT2 epitope of VLPs to the OT2 CD4^+^ T cells. CFSE‐labelled CD4^+^ T cells from naive OT2 mice were co‐cultured either with DCs (Fig. 2b) or with freshly isolated B cells from wild‐type BL6 mice (Fig. 2c) or Env BCR‐specific b12 mice (Fig. 2d) or HEL BCR‐specific SW‐HEL mice (Fig. 2e) in the presence of different VLPs (Fig. 2d,e; Table 1). OT2 CD4^+^ T cells proliferated equally well in the DC co‐cultures after stimulation with Env‐OT2‐VLPs and HEL‐OT2‐VLPs (Fig. 2b). In contrast, B cells only induced proliferation of the OT2 CD4^+^ T cells if the VLPs with OT2 epitope carried a surface antigen that matches the BCR specificity (Fig. 2c–e). Hence, only BCR‐dependent uptake of VLPs leads to efficient processing and presentation of the OT2 epitope by B cells and induces proliferation of OT2‐specific CD4^+^ T cells.

### Generation Tfh‐like cells from TCR‐transgenic naive CD4^+^ T‐cell precursors *in vitro* requires BCR‐dependent uptake of the VLPs

**Figure 3:**
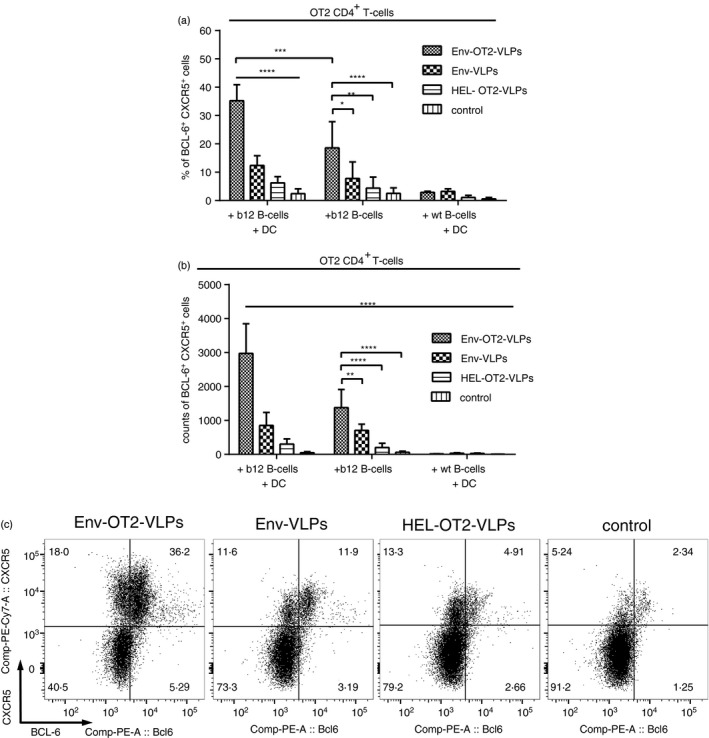
Generation of follicular helper T (Tfh) ‐like cells from naive OT2 CD4^+^ T cells *in vitro*. CD4^+^ T cells from naive OT2 mice, B cells from naive b12 mice or wild‐type (wt) BL6 mice and/or splenic dendritic cells (DCs) from naive BL6 mice were co‐cultured in the presence of different virus‐like particles (VLPs). After 6 days of incubation, cells were stained for surface B220, CD4, CXCR5 and intracellular BCL‐6. The gating strategy is presented in the Supplementary material (Fig. S3). (a) Percentage of CD4^+^ CXCR5^+^ BCL‐6^+^ cells among total B220^−^ CD4^+^ cells. (b) Counts per of CD4^+^ CXCR5^+^ BCL‐6^+^ cells per time package (represent absolute values). (a, b) The histograms represent the mean from 11 independent experiments ± standard deviation. *p < 0·05; **p < 0·01; ***p < 0·001; ****p < 0·0001 one‐way analysis of variance with Tukey's post test. (c) Expression of BCL‐6 (intracellular) and CXCR5 (surface) is representatively shown for B220^−^ CD4^+^ cells that were incubated for 6 days with freshly isolated b12 B cells and wt DCs in the presence of different VLPs.

To test the requirement of a cognate epitope for differentiation of naive CD4^+^ T cells into Tfh‐like cells within our *in vitro* system, we co‐cultured OT2 CD4^+^ T cells with DCs and b12 B cells from naive animals in the presence of either Env‐OT2‐VLPs or Env‐VLPs (Fig. 3). After 6 days, a significant proportion of the CD4^+^ T cells co‐expressing both BCL‐6 and CXCR5 were detected in the cultures incubated with Env‐OT2‐VLPs (Fig. 3).

Replacing b12 B cells with B cells from wt BL6 mice or using HEL‐OT2‐VLPs instead of Env‐OT2‐VLPs abolished the generation of BCL6^+^ CXCR5^+^ CD4^+^ T cells (Fig. 3). Additionally, co‐culturing of the OT2 CD4^+^ T cells with DCs and b12 B cells in the presence of soluble, monovalent recombinant Env protein (gp120) together with the soluble OT2 peptide instead of Env‐OT2‐VLPs also did not result in generation of BCL6^+^ CXCR5^+^ CD4^+^ T cells (data not shown). These data indicate that stimulation of the B cells by BCR cross‐linking and/or BCR‐dependent uptake of the VLPs for antigenic presentation is crucial for the induction of Tfh‐cell differentiation *in vitro*. Beside the master transcriptional factor BCL‐6, Env‐OT2‐VLPs drove the differentiation of naive OT2 CD4^+^ T cells mainly into T‐bet‐expressing cells (see Supplementary material, Fig. S4). No increase in expression of RoR*γ*t and Foxp3 was observed (data not shown).

When OT2 CD4^+^ T cells were co‐cultured with b12 B cells in the absence of DCs and stimulated with Env‐OT2‐VLPs, generation of the BCL‐6^+^ CXCR5^+^ CD4^+^ T cells was significantly reduced, but not abolished suggesting an auxiliary role of DCs during the differentiation process (Fig. 3a,b).

### Phenotypic and functional characterization of the Tfh‐like cells generated from OT2 CD4^+^ T‐cell progenitors

**Figure 4:**
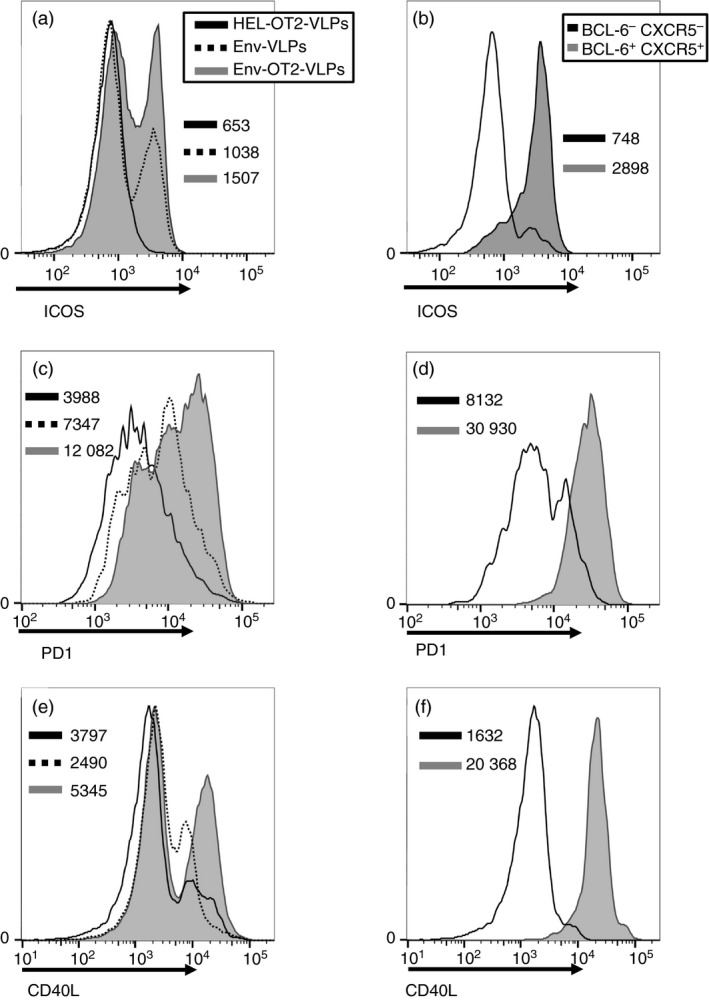
Phenotypic characterization of OT2 follicular helper T (Tfh) ‐like cells. (a, c, e) Histograms represent surface expression of ICOS (a), PD‐1 (c) and CD40L (e) on total population of OT2 CD4^+^ T cells from 6‐day co‐cultures with naive b12 B cells and dendritic cells (DCs) in the presence of different virus‐like particles (VLPs). (b, d, f) Histograms represent surface expression of ICOS (b), PD1 (d) and CD40L (f) on two different subpopulations of OT2 CD4^+^ T cells from 6‐day co‐cultures with naive b12 B cells and DCs in the presence of Env‐OT2‐VLPs. (a–f) Numbers specify geometric fluorescent mean of corresponding cell populations. Each experiment was performed four times, one representative experiment is shown. The gating strategy is shown in the Supplementary material (Fig. S3).

**Figure 5:**
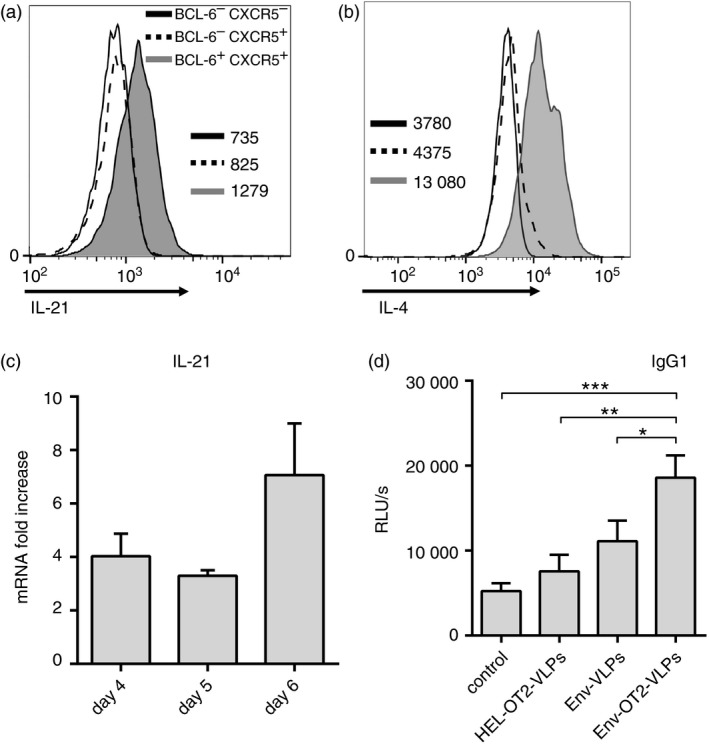
Functional characterization of OT2 follicular helper T (Tfh) ‐like cells. Untouched CD4^+^ T cells from naive OT2 mice were co‐cultured with freshly isolated b12 B cells and wild‐type (wt) dendritic cells (DCs) in the presence of different virus‐like particles (VLPs). (a, b) Histograms represent intracellular expression of interleukin‐21 (IL‐21) (a) and IL‐4 (b) among subpopulations of OT2 CD4^+^ T cells from co‐cultures in the presence of Env‐OT2‐VLPs. Numbers specify geometric fluorescent mean of corresponding cell populations. The data of one representative experiment out of four are shown; the gating strategy is shown in the Supplementary material (Fig. S3). (c) RT‐qPCR analysis of IL‐21 mRNA in the bulk RNA on days 4, 5 and 6 of the culturing with Env‐OT2‐VLPs. Expression of IL‐21 is shown as the fold increase over unstimulated controls after normalization to housekeeping genes. The histograms represent the mean values from three independent experiments ± standard deviation. (d) Supernatants from 6‐day cultures were analysed by ELISA for anti‐Env IgG1. The diagrams represent the mean values from seven independent experiments ± standard deviation. *p <* *0·05; **p <* *0·01; ***p <* *0·001 one‐way analysis of variance with Tukey's post test.

The surface expression levels of the molecules associated with Tfh cells were generally increased on CD4^+^ T cells after co‐culture with b12 B cells and DCs in the presence of Env‐OT2‐VLPs in contrast to the other VLP types (Fig. 4a,c,e). Phenotypic characterization of the BCL‐6 and CXCR5 double‐positive CD4^+^ T cells confirmed high expression levels of the molecules associated with conventional Tfh cells such as ICOS, PD1 and CD40L (Fig. 4b,d,f). In addition to phenotypic markers, detectable intracellular production of IL‐21 (Fig. 5a) and IL‐4 (Fig. 5b) was observed in BCL6^+^ CXCR5^+^ CD4^+^ T cells by intracellular cytokine staining. The induction of IL‐21 expression in the co‐cultures was also confirmed on the mRNA level (Fig. 5c). The helper function of the generated Tfh cells was also indirectly confirmed by the pronounced IgG1 class‐switch in the anti‐Env antibody production by the transgenic b12 B cells (Fig. 5d).

Hence, our study presents a robust *in vitro* experimental system for efficient generation of functionally active Tfh cells and confirms the importance of cognate B‐ and T‐cell cross‐talk for the Tfh differentiation process.

## Discussion

Current protocols for *in vitro* generation of Tfh‐like cells are limited to TCR‐transgenic T cells. Antigen‐specific stimulation of the CD4^+^ T cells by peptide‐loaded antigen‐presenting cells requires an additional supply of exogenous cytokines and anti‐cytokine antibodies.^4^, ^5^ In contrast to previous attempts, we used VLPs to mimic the natural antigenic stimulation of cognate B cells. Previously, we demonstrated the efficient antigen‐dependent activation and differentiation of naive B cells from SW‐HEL BCR‐transgenic mice with the cognate VLPs.^7^, ^9^ Such HIV‐derived HEL‐VLPs (Table 1) carry HEL as a transmembrane protein on the surface of the particles in a repetitive manner to mimic the viral envelope protein.^7^ As a B‐cell targeting antigen‐delivery system, these HEL‐VLPs are able to provide several unique direct effects on the cognate B cells that cannot be achieved by the monovalent form of the antigen.^7^, ^9^

Although the stimulation of B cells clearly depends on the HEL antigen,^7^ the antigen specificity of the subsequent interaction of the B cells with wt CD4^+^ T cells in the co‐culture experiments from Fig. 1 was not demonstrated and the antigen specificity of the generated wt Tfh cells was unclear. HEL is unlikely as a source of CD4 epitopes, because CD4^+^ T cells from BL6 mice are genetically ‘non‐responsive’ to HEL.^13^ However, VLPs as well as exosomes incorporate host proteins from the human HEK293T producer cell line providing a wide array of potential T helper cell epitopes. We previously demonstrated that HEL‐VLPs can effectively cross‐link BCR on naive SW‐HEL B cells, leading to their activation and rapid HEL‐VLP internalization.^7^ Subsequently, SW‐HEL B cells should be able to present peptides derived from HEL as well as from viral and cellular proteins of the VLP on their MHC‐II molecules. Therefore, effective interaction of these VLP‐activated, antigen‐presenting SW‐HEL B cells with CD4^+^ T cells specific for any of the VLP‐associated cellular and viral proteins is feasible.^6^ Based on a doubling time of antigen‐specific CD4 cells of 11 hr,^14^ a single T cell specific for one of the multiple epitopes in our starting population of 2 × 10^5^ CD4^+^ T cells could have a more than 8000‐fold expansion over the 6‐day culture period. At the same time, T cells lacking specificity for a VLP‐associated epitope die and further increase the percentage of the antigen‐specific Tfh cells. Therefore, the frequency of CD4^+^ T cells specific for one of the VLP‐associated epitopes seems to be high enough even in a naive wild‐type T‐cell population to result in a Tfh cell frequency of 10% after a 6‐day expansion period.

To answer the question, whether or not the Tfh cells from our *in vitro* system could be specific for other components of VLPs, we had to employ a TCR‐transgenic mouse model. As TCR‐transgenic mice for HIV‐1 proteins are not available, we adapted the OT2 CD4^+^ T cells by incorporating the OT2 epitope into the structural HIV‐1 GagPol protein of the VLPs (Fig. 3a; Table 1). To confirm our results obtained in the SW‐HEL mouse model with a vaccine‐relevant antigen, we introduced BCR‐transgenic B cells from b12 mice^12^ into the system. Therefore we were able to use Env‐VLPs that are identical to the conventional HIV‐VLPs (Table 1).

Generation of Tfh cells *in vivo* is thought to be initiated and driven by DCs.^1^, ^2^ Although signals from cognate activated B cells have been shown to play an important role in Tfh cell formation, the absolute requirement for B cells is questioned.^3^ Recent data from Trüb *et al*.^15^ demonstrate, however, that the Tfh differentiation pathway is initially B‐cell‐independent, then dependent on non‐cognate B‐cell interactions, and finally following cognate interaction with B cells and CXCR5‐ligands allows the formation of PD1^hi^ germinal centre Tfh cells.

In our double‐transgenic *in vitro* co‐culture system for the most efficient differentiation into Tfh cells, naive OT2 T cells require TCR‐mediated interaction with both DCs and the b12 B cells (Fig. 3). Lack of DCs only reduced, but did not abolish, Tfh differentiation, if the b12 B cells were still able to present the OT‐2 epitope from Env‐OT2‐VLPs (Fig. 2d). On the other hand, DCs presented the OT2 peptide of VLPs independently of the surface protein of the VLPs (Fig. 2b), but after incubation with HEL‐OT2‐VLPs b12 B cells did not (Fig. 2d) and, consequently, there was no generation of BCL‐6^+^ CXCR5^+^ T cells in the co‐culture (Fig. 3). The multi‐component concept of the *in vivo* Tfh differentiation process^1^, ^2^, ^3^, ^11^ with a leading role of DCs and an optional participation of cognate B cells is mainly based on the usage of monovalent proteins as B‐cell antigens. In contrast to monovalent proteins, VLPs are superior activators of cognate B‐cell responses. VLPs cross‐link BCRs^7^ and contain potential Toll‐like receptor stimuli,^16^ which may contribute to their efficient B‐cell activation and differentiation in cell culture^7^ and their immunogenicity *in vivo*.^9^, ^17^ Replacing Env‐OT2‐VLPs with a combination of monomeric HIV‐Env protein and the OT‐2 peptide did not induce the generation of BCL‐6^+^ CXCR5^+^ T cells as well (data not shown). Although the OT‐2 peptide alone was sufficient to induce an initial proliferative response of OT2 CD4^+^ T cells, the Env protein as a cognate monovalent antigen is not able to cross‐link BCR like cognate VLPs do.^7^ Taken together, the BCR‐dependent stimulation with cognate VLPs and the presentation of the OT2 epitope by the B cells are the critical parameters triggering the differentiation of the OT2 CD4^+^ T cells into Tfh cells *in vitro*.

Using a variety of bone marrow chimeric mice, Trüb *et al*.^15^ defined multiple steps in the Tfh differentiation pathway that have distinct molecular requirements and may differ in phenotype and function depending on environmental cues. *In vitro* generated wt (Fig. 1) and OT2 (Figs 3 and 4) PD1^hi^ BCL‐6^+^ CXCR5^+^ CD4^+^ Tfh cells have similarities with *in vivo* CXCR5^+^ PD1^hi^ Tfh cells described by Trüb *et al*.: generation of both cell types requires cognate interactions with B cells.^15^ Interleukin‐4 production by *in vitro* generated PD1^hi^ BCL‐6^+^ CXCR5^+^ CD4^+^ Tfh cells also has similarities with mature germinal centre Tfh cells generated after lymphocytic choriomeningitis virus infection in mice.^11^ Yusuf *et al*. showed that the main source of IL‐4 for the B‐cell development in germinal centres were the Tfh cells. Interestingly, the expression of IL‐4 by the germinal centre Tfh population was Th2 independent, because GATA3 expression, as the signature transcription factor for Th2 cells, was not increased above basal levels in any virus‐specific CD4 T cells, and other Th2 cell‐associated mediators were undetectable.^11^ At the same time, the majority of virus‐specific non‐Tfh CD4^+^ T cells and most of the non‐germinal centre Tfh cells produced IFN‐*γ*.^11^ Our *in vitro* experimental data (Fig. 5b and see Supplementary material, Fig. S4) are consistent with this *in vivo* observation.

Some BCL‐6^low/–^ CXCR5^+^ CD4^+^ cells that we also observed in our cultures (Figs 1 and 3) might correspond to the earlier stage of Tfh development *in vivo* (cognate B‐cell‐independent precursors of PD1^hi^ Tfh cells).^15^ However, in contrast to the situation *in vivo* 1, 2, 15 we detected these cells also in the absence of DCs. Since *in vivo* naive CD4^+^ T and B cells have distinct histological locations within the secondary lymphoid organs, antigen‐activated B cells have no access to the T‐cell zones to provide naive cognate CD4 T cells with appropriate signals.^2^, ^18^ Additionally, naive antigen‐specific B cells in wt animals are exceedingly rare and, therefore, might not be available as the initial antigen‐presenting cells for CD4^+^ T cells during responses to infections or protein immunizations.^2^ Within the *in vitro* cultures, however, antigen‐naive CD4^+^ T cells have direct cognate contact with numerous BCR‐transgenic B cells that are activated by VLPs in an antigen‐specific manner. This may reduce the absolute requirement for directive instructions from DCs observed *in vivo*.

Hence, the results from our *in vitro* study are consistent with the multi‐component concept. However, it switches accepted roles and responsibilities: the cognate B‐ and T‐cell interaction seems to play a crucial role, at least when VLPs are used as a trigger *in vitro* (Figs 1 and 3).

Our study presents a reliable versatile platform for the generation of functional Tfh cells *in vitro* and confirms the importance of B‐ and T‐cell cognate cross‐talk for the Tfh differentiation process.^15^ Moreover, the study confirms that VLPs are unique activators of cognate B cells with an impact on T helper cell differentiation.^6^, ^7^, ^9^ Simplicity, natural biological background and minimal additional requirements make this model relevant for both basic scientific research on Tfh cell biology and rational vaccine design.^6^

## Disclosures

The authors declare no commercial or financial conflicts of interest.

## Supporting information


**Figure S1**. Gating strategy for Fig. 1(a).

**Figure S2.** Gating strategy for Fig. 2(b–e).

**Figure S3.** Gating strategy for Figs 3, 4 and 5(a,b).

**Figure S4.** Expansion of GATA3 and T‐bet positive CD4^+^ T cells.
