# Supplementary figures and images for: Generation of T follicular helper cells in vitro: requirement for B‐cell receptor cross‐linking and cognate B‐ and T‐cell interaction
Source: Immunology. 2017 Oct 9;153(2):214–24. doi: 10.1111/imm.12834 (PMC5765376; doi:10.1111/imm.12834)

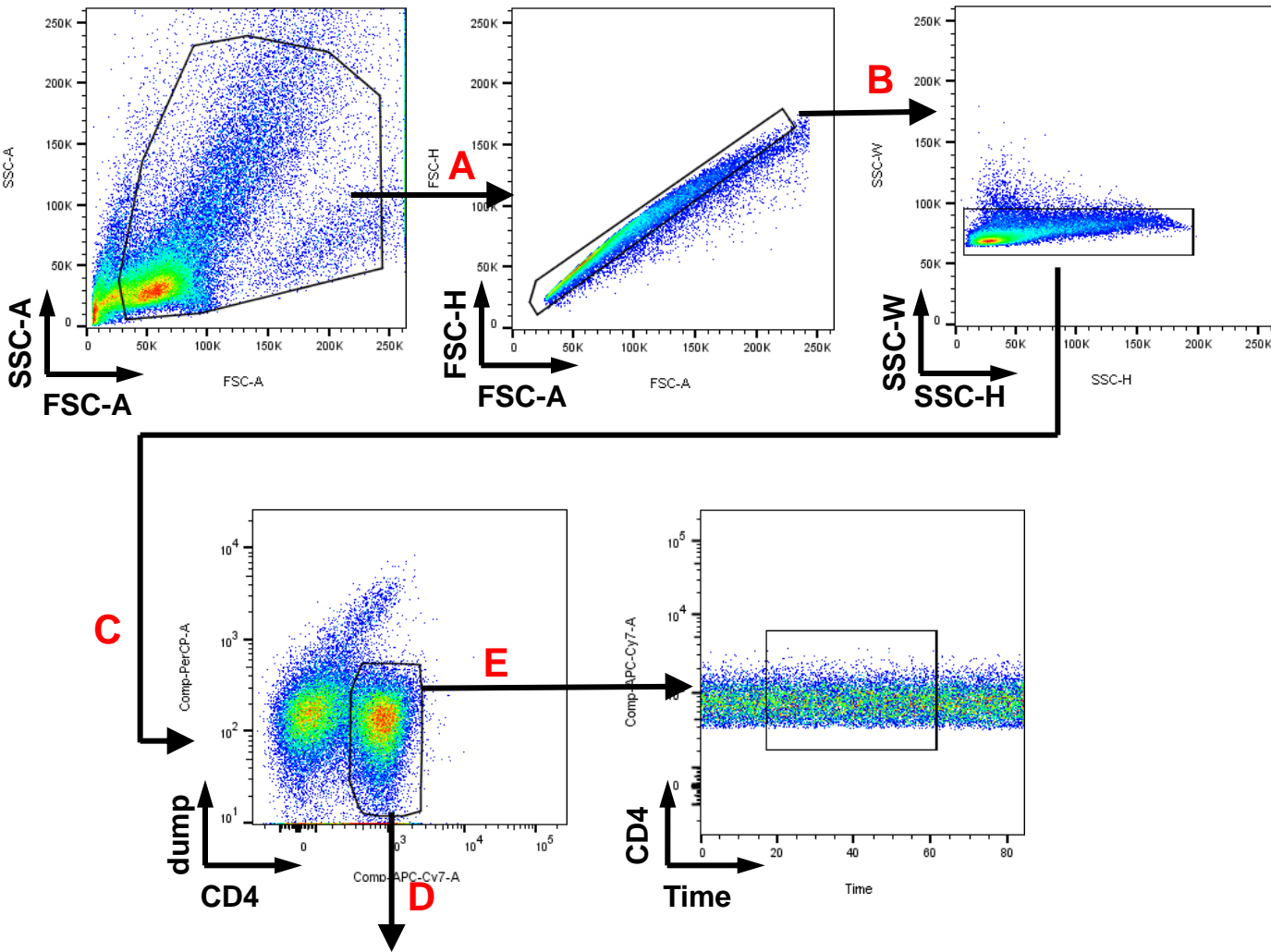

Figure 1A

Supplement: Supplementary file 1 — Figure S1. Gating strategy for Fig. 1(a). [file IMM-153-214-s001.pdf]

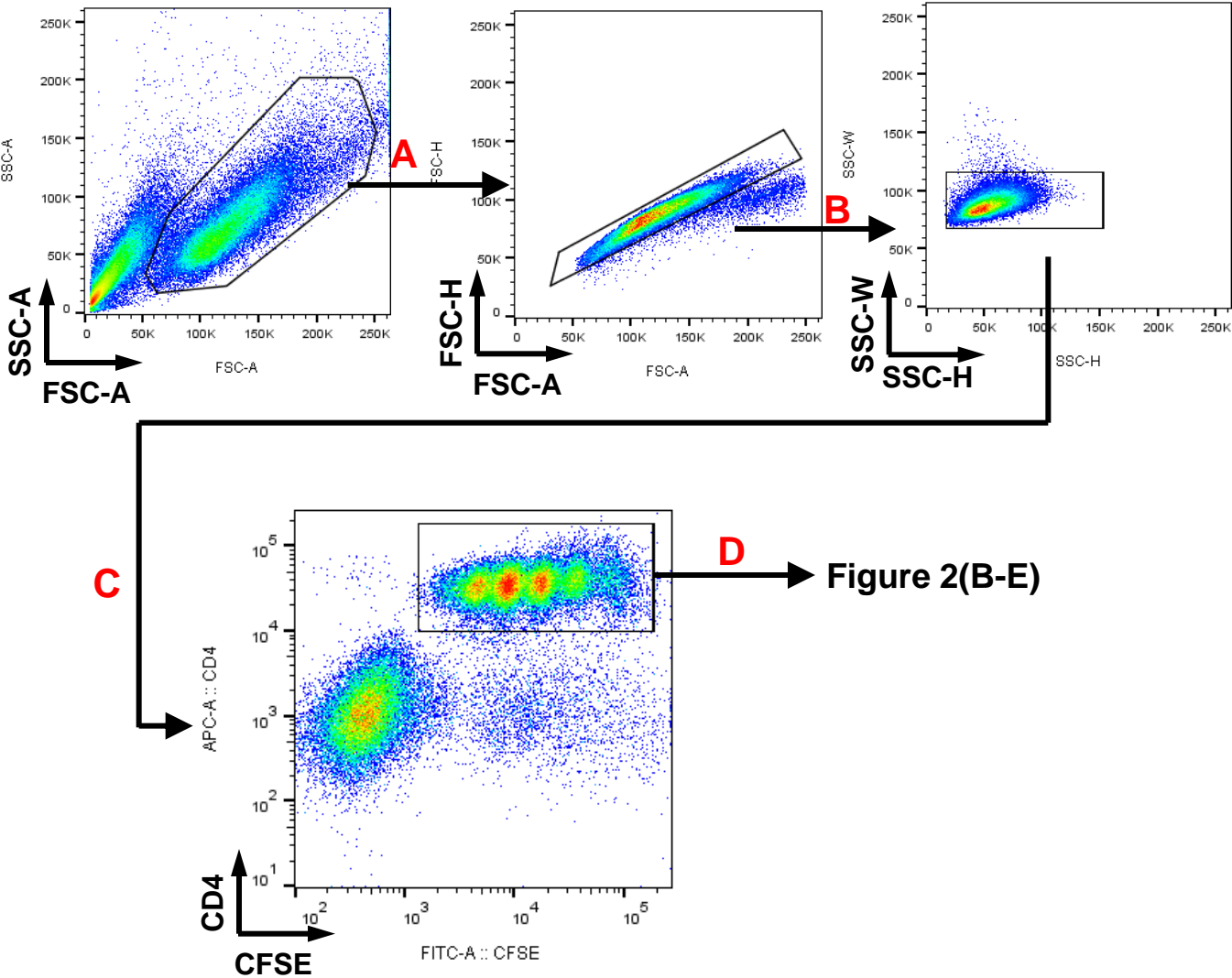

Supplement: Supplementary file 2 — Figure S2. Gating strategy for Fig. 2(b–e). [file IMM-153-214-s002.pdf]

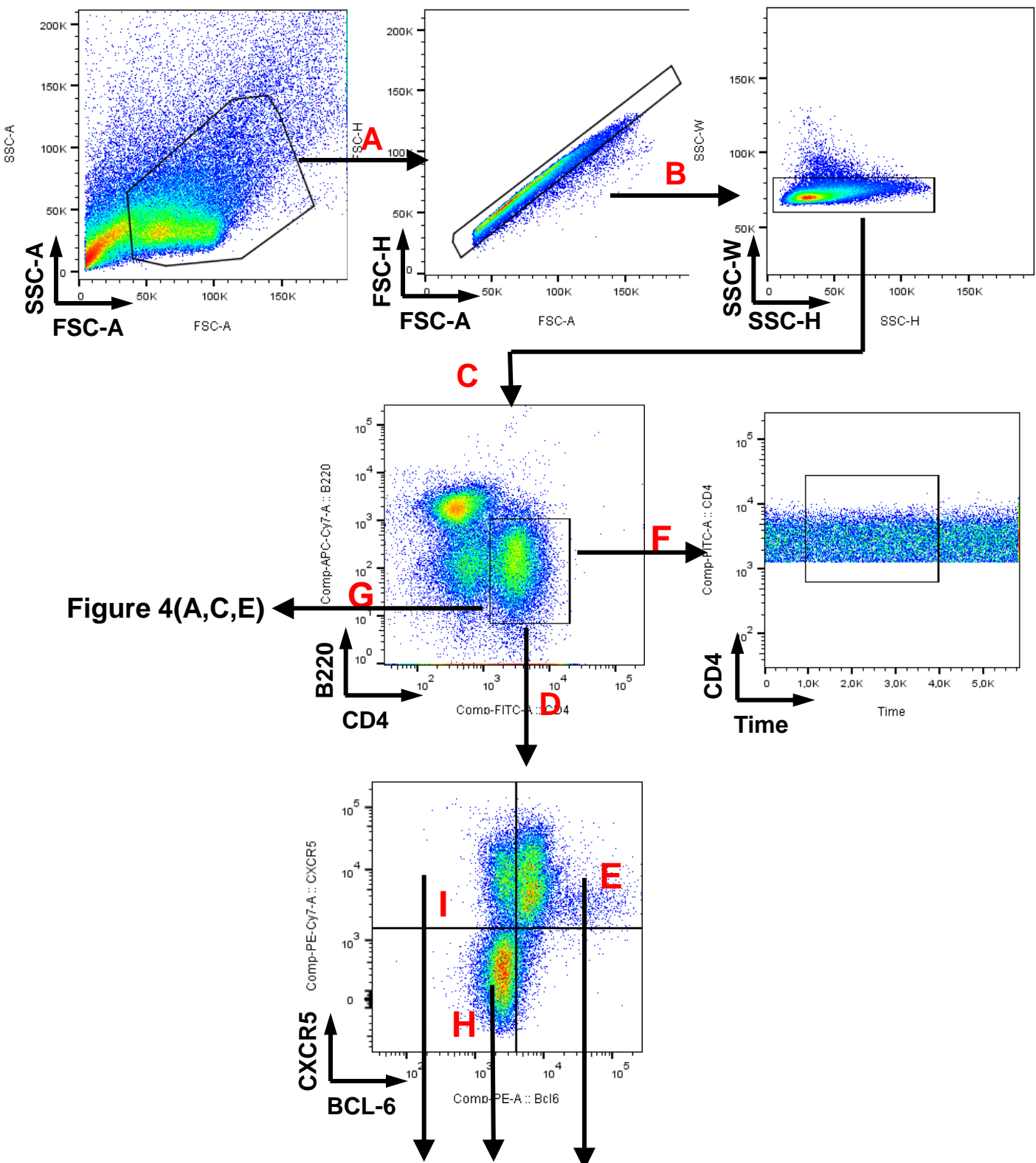

Figure 4(A,C,E)

Figure 3A, Figure 4(B,D,F) and Figure 5(A,B)

Supplement: Supplementary file 3 — Figure S3. Gating strategy for Figs 3, 4 and 5(a,b). [file IMM-153-214-s003.pdf]

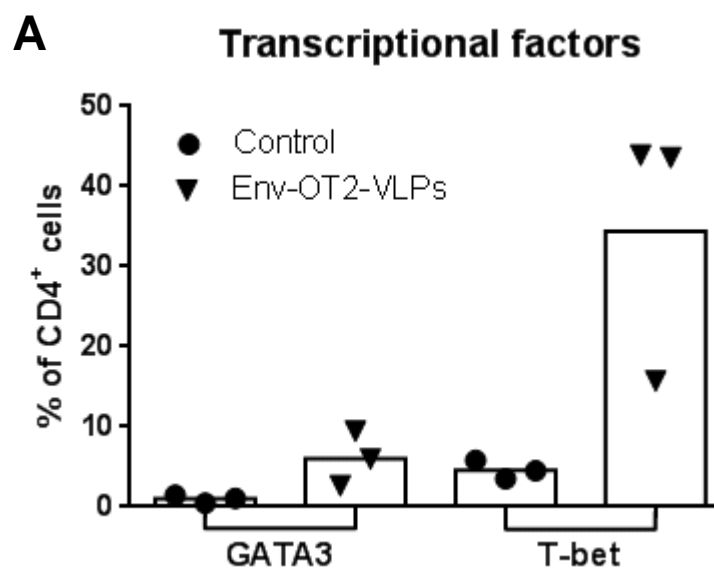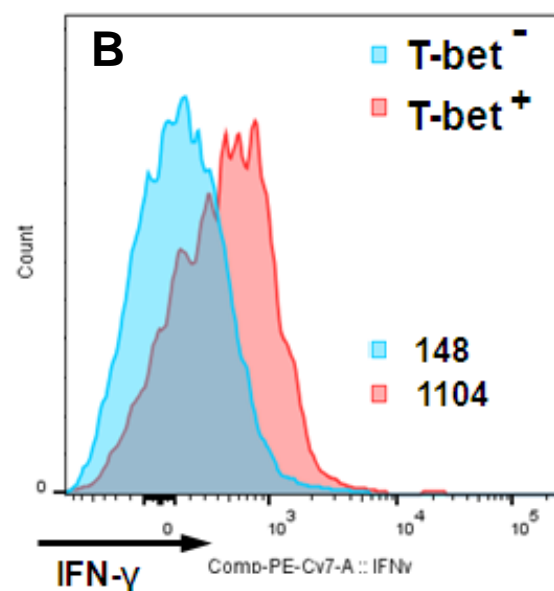

Supplement: Supplementary file 4 — Figure S4. Expansion of GATA3 and T‐bet positive CD4+ T cells. [file IMM-153-214-s004.pdf]
