# Supplementary material for: Generation of T follicular helper cells in vitro: requirement for B‐cell receptor cross‐linking and cognate B‐ and T‐cell interaction
Source: Immunology. 2017 Oct 9;153(2):214–24. doi: 10.1111/imm.12834 (PMC5765376; doi:10.1111/imm.12834)
Supplement: Supplementary file 5 [file IMM-153-214-s005.docx]

**Supplemental Figure Legends**

**Figure S1. Gating strategy for Figure 1A.**

After gating on lymphoid cells according to their forward and sideward scatter (**A**) doublets were discriminated using both FSC-H vs. FSC-A (**B**) and SSC-H vs. SSC-W (**C**) gating strategies. A dump channel was used to exclude auto-fluorescent cells during the selection of CD4 positive cell singlets (**D**, **E**). (**D**) CD4 positive cell singlets were then analyzed for the expression of BCL-6 (intracellular) and CXCR5 (surface) as it is representatively showed in **Figure 1A**. (**E**) The probe flow cytometry measurements were performed automatically using the BD FACS Loader, therefore gating on a time period (time package) was used to analyze cell counts (i.e. absolute number of CD4 positive cells) per time package. Cell counts for BCL-6/CXCR5 subpopulation were calculated individually according the percentage of corresponding subpopulation among the total CD4^+^ cells.

**Figure S2. Gating strategy for Figure 2B-E.**

After gating on lymphoid cells according to their forward and sideward scatter (**A**) doublets were discriminated using both FSC-H vs. FSC-A (**B**) and SSC-H vs. SSC-W (**C**) gating strategies. (**D**) CFSE and CD4 positive cell singlets were selected for further demonstration on CFSE-histograms in **Figure 2B-E**. Pseudocolor plots demonstrate representative flow cytometric analysis of CFSE labelled OT2 CD4^+^ T-cells incubated with b12 B-cells in the presence of Env-OT2-VLPs.

**Figure S3. Gating strategy for Figure 3, Figure 4, and Figure 5A-B.**

After gating on lymphoid cells according to their forward and sideward scatter (**A**) doublets were discriminated using both FSC-H vs. FSC-A (**B**) and SSC-H vs. SSC-W (**C**) gating strategies. B220 negative and CD4 positive cell singlets were selected for further analysis (**D-I**). (**D**) Expression of BCL-6 (intracellular) and CXCR5 (surface) on B220-CD4+ cells were used in **Figure 3C**. (**E**) Percentages of B220^-^CD4^+^BCL-6^+^CXCR5^+^ among total B220^-^CD4^+^ singlet cells under different culture conditions were summed up in **Figure 3A**. (**F**) Gating on a time period (time package) was used to analyze cell counts (represent absolute values) per time package. Cell counts subpopulation were calculated individually according the percentage of corresponding subpopulation among the total CD4^+^ cells and were summed up in **Figure 3B**. Surface expression of ICOS, PD-1, and CD40L on total population of OT2 B220^-^CD4^+^ cells (**G**), or on B220^-^CD4^+^BCL-6^+^CXCR5^+^ (**E**) and B220^-^CD4^+^BCL-6^-^CXCR5^-^ (**H**) subpopulations were further demonstrated on the corresponding histograms of **Figure 4**. Intracellular cytokines expression for subpopulations (**E**)**,** (**H),** and (**I**) were demonstrated in **Figure 5A-B.**

**Figure S4. Expansion of GATA3 and T-bet positive CD4^+^ T-cells.**

CD4^+^ T-cells from naïve OT2 mice, B-cells from naïve b12 mice and splenic DCs from naïve BL6 mice were co-cultured in the presence of Env-OT2- VLPs. After 6 days of incubation, cells were stained for surface B220, CD4 and intracellular for GATA3 or T-bet and IFN-gamma. **(A)** Percentage of GATA3 and T-bet expressing cells among total B220^-^CD4^+^ cells in comparison to the control group. The histograms represent the mean values from 3 independent experiments and the individual experiment values. **(B)** Intracellular expression of T-bet IFN-gamma among T-bet positive and negative subpopulations of OT2 B220^-^CD4^+^ T-cells from co-cultures in the presence of Env-OT2-VLPs. Numbers specify geometric fluorescent mean of corresponding cell populations. The data of one representative experiment out of three are shown.
